# Effect of Standardized Yelling on Subjective Perception and Autonomic Nervous System Activity in Motion Sickness

**DOI:** 10.3390/ijerph182312854

**Published:** 2021-12-06

**Authors:** Min-Yu Tu, Hsin Chu, Chung-Yu Lai, Kwo-Tsao Chiang, Chi-Chan Huang, Hsien-Chuan Chin, Yu-Hsin Wen, Chien-Liang Chen

**Affiliations:** 1 Aviation Physiology Research Laboratory, Kaohsiung Armed Forces General Hospital Gangshan Branch, Kaohsiung 820, Taiwan; du0807@yahoo.com.tw (M.-Y.T.); charco66@gmail.com (K.-T.C.); pp810020@814.afgsh.org.tw (C.-C.H.); mutation@814.afgsh.org.tw (H.-C.C.); allwen@gmail.com (Y.-H.W.); 2 Department of Life Sciences and Ph.D. Program in Translational Medicine, National Chung Hsing University, Taichung 402, Taiwan; 3 Department of Health Business Administration, Meiho University, Pingtung 912, Taiwan; 4 Institute of Medical Science and Technology, National Sun Yat-sen University, Kaohsiung 804, Taiwan; 5 Medical Section, Civil Aviation Medical Center, Taipei City 105, Taiwan; hrchu@mail.ndmctsgh.edu.tw; 6 Graduate Institute of Aerospace and Undersea Medicine, National Defense Medical Center, Taipei City 114, Taiwan; multi0912@gmail.com; 7 School of Public Health, National Defense Medical Center, Taipei 114, Taiwan; 8 Department of Business Management, National Sun Yat-sen University, Kaohsiung 804, Taiwan; 9 Department of Physical Therapy, I-Shou University, Yan-chao District, Kaohsiung 824, Taiwan

**Keywords:** yelling with abdominal force, vocalization, heart rate variability, cognitive responses, Coriolis stimulation

## Abstract

This study investigated the effects of yelling intervention on symptoms and autonomic responses in motion sickness. Forty-two healthy participants were recruited, and they participated in Coriolis stimulation, a technique for inducing motion sickness. The experimental procedure comprised five 1-min rotating stimuli with 1-min rest after each stimulus. Then, the symptom severity was assessed using the Motion Sickness Symptom Rating (MSSR). The d2 Test of Attention scores and cardiovascular responses were recorded before and after Coriolis stimulation. The electrocardiogram results were documented to analyze heart rate variability (HRV). During Coriolis stimulus, the participants were required to yell 5–8 times in the experimental trial, and to keep quiet for each minute of rotation in the control trial. The yelling intervention significantly reduced the MSSR score (*p* < 0.001). Nevertheless, it did not significantly affect the d2 Test of Attention scores. Yelling while rotating did not significantly affect the heart rate nor blood pressure. However, it decreased the normalized low frequency of HRV (*p* = 0.036). Moreover, it improved motion sickness, but its effect on attention was not evident. Motion sickness could significantly affect cardiovascular responses and HRV. However, yelling did not affect cardiovascular response, and it reduced sympathetic nervous system activity.

## 1. Introduction

Motion sickness can be classified into vehicle motion sickness (such as seasickness and airsickness), amusement-park-ride sickness, and Cinerama sickness caused by different stimuli. Currently, these conditions can be explained by the sensory conflict or neurological mismatch hypothesis proposed by Claremont C.A. in 1931 [1]. Generally, motion sickness is caused by contradictory movement information received from the eyes and other sense organs (vestibular and proprioceptors) [2]. If dynamic or visual stimulation is strong or long enough, most people will experience maladaptive motion sickness. Motion sickness symptoms include pallor, cold sweats, nausea, dizziness, headache, and vomiting. The cause of motion sickness signs and symptoms is still not fully elucidated. This condition leads to a dynamic imbalance of sympathetic and parasympathetic nervous system activities under the brainstem. This notion may be supported by the mechanism of action of two commonly used anti-sickness medications (parasympathetic inhibitors and sympathetic stimulants) [3,4]. Electrocardiogram (ECG) is sensitive to autonomic dysfunction. Changes in the ECG-derived heart rate variability (HRV) are the biomarker of the sympathetic and parasympathetic activity. It is supposed to be a useful tool for identifying motion sickness or other disease [5,6,7].

Although motion sickness does not directly threaten lives, its impact varies. That is, its effect on personal travel is not serious. However, that on competition performance is more important. Further, its impact on the combat capability and flight safety of air force pilots is the most dangerous. Nevertheless, most motion sickness medications have side effects, such as drowsiness, blurred vision, and attention disturbance. Moreover, these medications must be taken 30 min before boarding the transportation (before symptom onset) [8,9]. The abovementioned medications have undesirable side effects. Thus, further research about non-pharmacologic measures for motion sickness symptoms must be conducted. Indeed, the side effects of these medications can be quite serious among flight attendants whose work require mental attention and vigilance. The motivation of this research was to find out an immediate and no side effect behavior strategy for the groups often affected by motion sickness (such as pilots, paratroopers, etc.) to help solve the problems of people who are susceptible to motion sickness, and reduce training costs. This study focused on whether yelling intervention can mediate or inhibit negative symptoms, which, in turn, can prevent the negative physiological and mental side effects of motion sickness medications.

Some studies have assessed the effects of a standardized yelling maneuver (referred to as the APRL maneuver in our laboratory) on space disorientation and exercise performance [10,11,12]. Results showed that this yelling maneuver could speed up the recovery of pilots from spatial disorientation. The main physiological mechanism was to increase the mean power frequency of electroencephalography (EEG), and to reduce electrooculogram activity, thereby improving the pilot’s ability to control the aircraft [11]. Other studies have also shown that stimulation of brain activity can affect the response of HRV [13,14]. In addition, Kotani et al., (2007) assessed the effect of vocalization on HRV, and found that speaking loudly could excite the sympathetic nerves, and decrease parasympathetic nervous system activity [15]. Therefore, we hypothesized that yelling (vocalization) can affect attention, and regulate the autonomic nervous system by increasing brain activity. The current study aimed to investigate the influence of the standardized yelling maneuver on Coriolis illusion-induced motion sickness. Moreover, to evaluate the potential of this intervention, subjective feelings, attention, and autonomic nervous system activity during motion sickness were assessed.

Motion sickness has always been one of the main reasons for the elimination of personnel when training particular athletes or occupations (for example, pilots, astronauts, ballet dancers). At present, non-pharmaceutical research is more concerned about the effect of acupuncture [16], but its application in dynamic task execution still has many limitations. Therefore, a yelling maneuver was developed to respond, which is simple and convenient, and has no occasional restrictions. If the yelling maneuver developed in this study can effectively improve the symptoms of motion sickness, and understand its physiological mechanism, it will be applicable to some people who cannot use anti-sickness drugs due to the characteristics of work or training (for example, pilot training, survival training at sea).

## 2. Materials and Methods

### 2.1. Participants

This study recruited 49 healthy volunteers, of which only 42 completed the whole rotating chair stimulation procedure, and 7 withdrew prematurely because they could not tolerate the symptoms of excessive motion sickness. The average age of the participants (male: *n* = 38; female: *n* = 4) was 26.6 ± 0.8 years old; height, 173.3 ± 1.0 cm; and weight, 71.1 ± 1.9 kg. The exclusion criteria included patients with gastrointestinal, cardiovascular, and vestibular system diseases. The participants were required to undergo a health examination within 1 year, and did not have evident abnormalities. Moreover, they must comply with experimental guidelines during the study period. These include not staying up late before the experiment, and not changing their daily routine. Further, they could not take drugs and beverages, including coffee and ginger tea, before the experiment. Informed consent was obtained from all participants. The study was conducted according to the guidelines of the Declaration of Helsinki, and it was approved by the Institutional Review Board of Kaohsiung Armed Forces General Hospital (protocol code: KAFGH_106-008).

### 2.2. Motion Sickness Induced by Coriolis Stimulation

The constant-speed rotation of the electric rotating chair (Lacidem, Taiwan) was used to stimulate the semicircular canals to produce a quantitative Coriolis stimulus, which is a technique used to induce motion sickness symptoms. The Coriolis illusion involved the simultaneous of two semicircular canals, and is associated with a sudden tilting (forward or backwards) of a person’s head while the rotating chair is turning. This can occur when participant has their head down (neck flexion), or if they tilt it up (neck extension) [17]. At the beginning of the experiment, the participants were instructed to sit quietly in a rotating chair for at least 5 min to help them calm down. During this period, basic physiological parameters (including blood pressure, heart rate, and electrocardiogram results) were assessed, and the d2 Test of Attention was performed. Then, the electric rotary chair was accelerated at a subthreshold rate for 30 s until a constant angular velocity of 120 degrees x s(−1) was reached. During each constant speed rotation, the participants were verbally instructed to tilt their heads up (neck extension) and down (neck flexion) along the pitch axis, and the total angle of movement was required to exceed 45 degrees [18]. Head movements need to reciprocate three times to induce evident motion sickness symptoms. The 10-min Coriolis stimulation process comprises five 1-min spin stimuli, with 1-min rest after each stimulus. During each rest period, the participants were verbally asked about subjective motion sickness symptoms, and the cumulative changes of motion sickness were assessed using the Motion Sickness Symptom Rating (MSSR).

### 2.3. Quantification of Motion Sickness Severity

After each 1-min rotation, the symptom severity was assessed using the MSSR. This scale was compiled by S. Klosterhalfen in accordance with the Graybiel diagnostic criteria [19]. The researchers verbally asked the participants’ scores for motion sickness, and the participants also responded verbally. The items evaluated using this scale included the seven typical symptoms of motion sickness, which are dizziness, headache, nausea, vomiting, fatigue, lethargy, cold sweats, and gastrointestinal discomfort. Scores of 3, 4, and 5 were used for the evaluation. When the score was higher, the motion sickness symptoms were stronger, and the highest total score was 35. To assess for recovery, the participants were evaluated again 10 min after the end of Coriolis stimulation.

### 2.4. Assessment of Individual Attention and Concentration Performance

In this study, the d2 Test of Attention (which is a standard paper-and-pen test) was used to measure a person’s attention and concentration [20]. It was performed before the spin stimulation, and 10 min after Coriolis stimulation (within the first minute of the recovery period). Moreover, it comprises 14 test lines in a horizontal arrangement, with 47 characters in each line. Each character consists of the letter “d” or “p,” marked with one, two, three, or four small dashes. The participants were instructed to scan the test lines, and delete all occurrences of the letter “d” with two horizontal lines, ignoring all other characters. Two types of errors were observed after completing the test, and these were missing errors (missing characters that should be deleted), and misjudgments (crossing out characters that should not be deleted). The test results include the following standard reference scores: the reliability of processing speed, which was measured by the total number of items processed ([TN], the sum of all items processed correctly or incorrectly); the percentage of errors ([E%], processed at all proportion of errors in projects), which was used to evaluate one’s quality performance; the total number of items processed minus the errors (TN-E), which was utilized to indicate the impact of attention on the combined scores of speed and accuracy; and the concentration performance ([CP], the number of correctly processed items minus the number of wrong judgments), which was utilized to measure the distortion of the response pattern. Before starting the study, all participants were completely trained on the d2 Test of Attention to minimize the effect of learning.

### 2.5. Evaluation of Autonomic Nervous System Activity

HRV is a measure of the degree of variation in each heartbeat interval (R–R interval in ECG), which reflects the balance of sympathetic and parasympathetic nervous system activities. In this experiment, a miniature physiological signal recorder (TD1, Taiwan Telemedicine Device Company, Taiwan) was used to capture the ECG of participants to analyze HRV for the basic value before rotation, changes during rotation, and recovery after 10 min of rotation. The raw ECG signals were recorded in real time after analog-to-digital conversion (8-bit) at a sampling rate of 250 Hz. The R–R intervals (in milliseconds) were calculated beat-to-beat using a customized software program developed by Dr. Terry B. J. Kuo [21]. The collected signals were subjected to a fast Fourier transform (FFT) in a frequency domain analysis to perform spectrum analysis. All the signals to be analyzed were truncated into successive 30-s epochs with 50% overlapping. A Hamming window was applied to each time segment to attenuate the leakage effect [22]. For each time segment (576 s, 4096 data points), our algorithm estimated the power spectral density based on FFT. The resulting power spectrum was corrected for attenuation resulting from sampling and the application of the Hamming window [21]. Each component of the spectrogram was subsequently quantified by the method of integration. The main analysis parameters included total power (0–0.4 Hz), very-low frequency power (VLF: 0.003–0.04 Hz), low-frequency power (LF: 0.04–0.15 Hz), high-frequency power (HF: 0.15–0.4 Hz), normalized LF (LF%), normalized HF (HF%), and low-to-high frequency (LF/HF) ratio. All HRV parameters were expressed in natural logarithmic form to demonstrate and correct possible skewness. Total power reflects autonomic nervous activity; HF power reflects parasympathetic nervous system activity; and LF power reflects partial contributions from both sympathetic and parasympathetic nervous system activities. In particular, LF was normalized by the percentage of total power except for VLF (total power—VLF) to detect sympathetic influence on HRV (LF%). A similar procedure was also applied to HF (HF%), and HF% reflects sympathetic inhibition. The LF/HF ratio was used as an index of sympathovagal balance [23,24].

### 2.6. Introduction of the Standardized Yelling Maneuver and the Time of Intervention

Immediately after each pitching head movement caused by the rotation-induced motion sickness, a standard abdominal yell was made with 5–8 sounds, which sounds like “Er.” The participant had to yell out with the abdominal muscles contracted. Each vocalization time was about 1 s. The yelling interval could be adjusted between 3 and 4 s, and the volume had to reach 100 decibels. The yelling time per round lasted about 20–30 s. The operation mode of the yelling maneuver was learned and practiced several times before the official start, to ensure the execution quality of the experiment.

Each participant had to perform the same experimental method and procedure twice (yelling trial vs. control trial). The difference was only whether the yelling maneuver was executed during the five 1-min rotations. The effects of the yelling intervention on subjective symptoms (as assessed using the MSSR and d2 Test of Attention) and objective physiological values (HRV, blood pressure, and heart rate) during motion sickness were recorded. These were used to compare the difference between the severity and physiological response to motion sickness when the yelling intervention was involved.

### 2.7. Experimental Procedure

**Figure 1:**
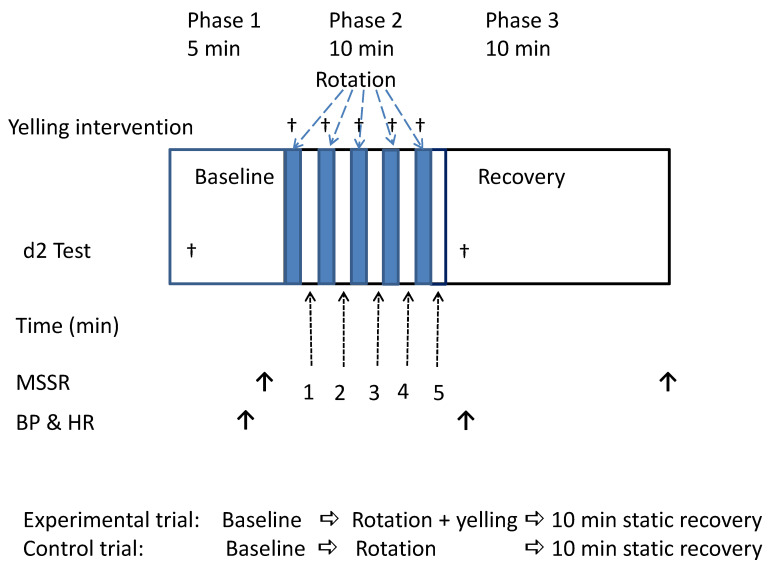
Experimental procedure and detection time of each parameter. BP = blood pressure; HR = heart rate; MSSR = Motion Sickness Symptom Rating.

Each participant underwent two rotating chair tests. The experimental trial (yelling trial) and the control trial were performed at the same time on different experimental days. The experimental and control trials only differed after the head movements of each rotation period were completed. That is, the participants of the experimental trial were instructed to perform 5–8 yelling maneuvers, whereas the participants of the control trial kept quiet, and did not do any movements. The two trials were performed at an interval of at least 2 days. However, to prevent interfering with the experimental results due to adaptive learning, the test order of the control and experimental trials was randomly arranged. Before the start of the experiment, we arranged a lottery to randomly determine half of the participants to intervene by yelling (experimental trial) for the first time in the two rotating tests; the remaining half of the participants yelled at the second time of the two tests. Figure 1 shows the detailed experimental procedure. The timing and purpose of each parameter in this study are described below.

### 2.8. Statistical Analysis

The severity of subjective motion sickness, and the participant’s attention function were assessed using the MSSR and the d2 Test of Attention scores, supplemented by HRV and other physiological response parameters as the objective assessment criteria. The total MSSR scores were ranked in order from the highest to the lowest, and the participants were divided into susceptible and non-susceptible groups for analysis.

The appropriate sample size was estimated using the G-Power 3.1.9.4 online program. A priori power analysis for repeated measures using an effect size of 0.45, power of 0.80, and α = 0.05 yielded a minimum sample size of *n* = 41. A repeated measured analysis of variance was used to analyze changes in MSSR scores five consecutive times during the spin stimulation. In addition, the d2 Test of Attention before and after rotation stimulation, pre- and post-test of physiological data, and changes during the 10-min recovery period were analyzed. The main effect of time, and the trial-by-time interaction between these trials significantly differed. The level of significance was set at *p* < 0.05, and tendency was noted for 0.05 < *p* < 0.09.

## 3. Results

### 3.1. Effect of the Yelling Intervention on the Severity of Motion Sickness Symptoms

**Figure 2:**
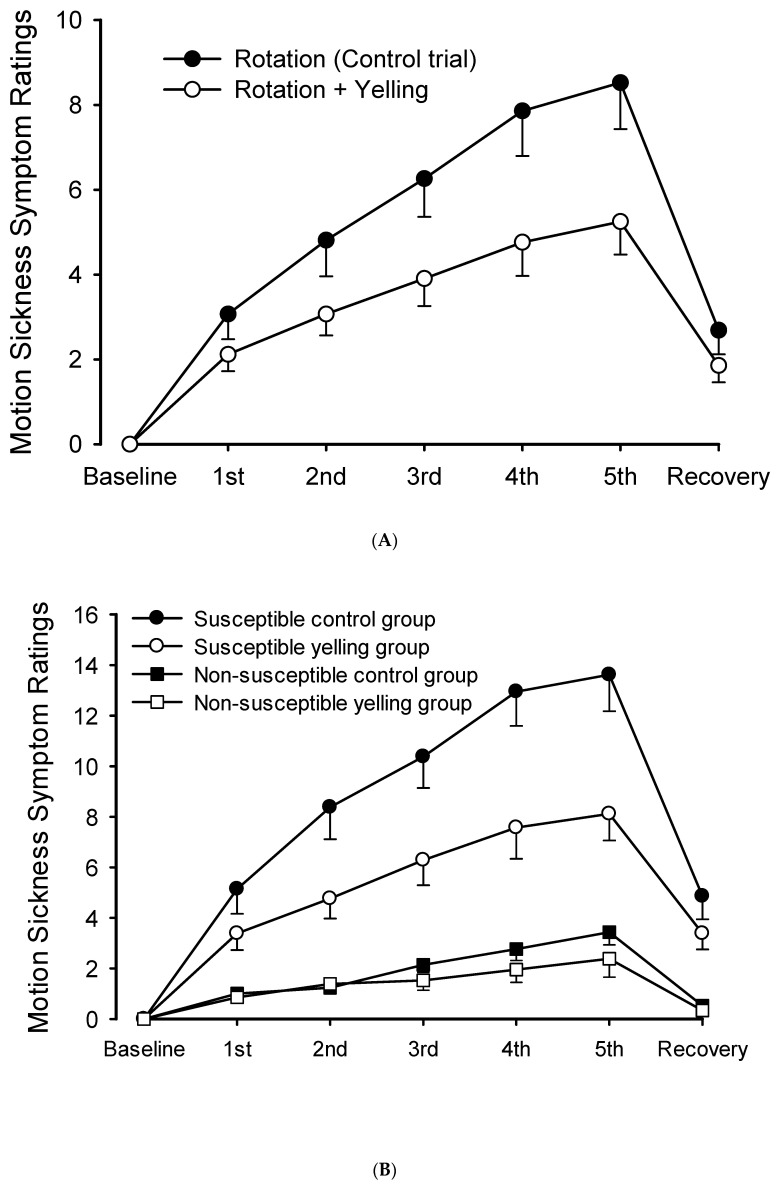
Scores of motion sickness symptoms during intermittent rotations and its recovery period. (**A**) The yelling intervention could significantly reduce motion sickness scores. (**B**) It significantly decreased motion sickness scores in the susceptible group. However, changes were not evident in the non-susceptible group.

The MSSR score continuously increased over time. However, it decreased significantly 10 min after recovery. The trial-by-time interaction did not significantly differ (F = 8.705, *p* < 0.001). The main effect of time on MSSR scores increased significantly (F = 51.002, *p* < 0.001). The MSSR score of participants in the yelling trial was significantly lower than that of participants in the control trial (F = 11.723, *p* = 0.001) (Figure 2A).

Moreover, the total scores of the five MMSRs (*n* = 42) were assessed, and the participants were classified into two groups (*n* = 21 people each). The half with the higher score was divided into the motion sickness susceptible group, and the half with the lower score was divided into the non-susceptible group. In the susceptible group, the yelling intervention significantly differed in terms of the trial-by-time interaction of MSSR scores during the rotation period (F = 7.961, *p* < 0.001). Similarly, the main effect of time (F = 66.429, *p* < 0.001), and the main effect of trials (F = 11.915, *p* = 0.003) significantly differed (Figure 2B). By contrast, the MSSR score of the non-susceptible group did not significantly differ in terms of trial-by-time interaction (F = 2.441, *p* = 0.108) nor the main effects of trials (F = 2.160, *p* = 0.157). However, the main effect of time significantly differed (F = 21.855, *p* < 0.001).

### 3.2. Effects of the Yelling Intervention on Cognition and Attention after Inducing Motion Sickness

The d2 Test of Attention was used to evaluate the effect of the yelling intervention on the after-effect performance of cognitive attention after rotation stimulation. Results showed that the attention performance scores (including TN, E%, TN-E, and CP) were significantly better than those of the pre-test values (main effect of time, *p* < 0.001). However, neither the trial-by-time interaction (*p* > 0.153) nor the between-trial effect (*p* > 0.479) significantly differed (data not shown).

We further analyzed the attention response of the susceptible and non-susceptible groups individually, and differences were observed between the two groups (Table 1). In the susceptible group, among all parameter responses of the d2 Test of Attention, only the main effects of time significantly differed (*p* ≤ 0.001), whereas the yelling intervention did not cause differences in attention response (main effects of trial, *p* ≥ 0.714), and there were no significant differences in terms of group-by-time interaction (*p* ≥ 0.205). Similarly, the statistical results of the d2 Test of Attention between the non-susceptible and susceptible groups were similar.

### 3.3. Effects of Simple Yelling on Cardiovascular and HRV at Rest

To determine the effect of pure yelling on physiological response at rest, 13 participants underwent the preliminary test at a time other than the experimental day. This test measured the heart rate, blood pressure, and 5-min HRV of the participants while at rest, and they were considered base values. Then, they were instructed to perform a standard yelling maneuver for 5 rounds for a total of 5 min. The conditions for executing the yelling maneuver were similar to phase 2 in Figure 1. However, the rotating chair was at a standstill during this period. Results showed that yelling significantly increased systolic blood pressure (*p* = 0.047) and diastolic blood pressure (*p* = 0.006). However, it had no significant effect on heart rate and all HRV parameters (Table 2).

### 3.4. Effects of the Yelling Intervention on Cardiovascular and Heart Rate Variability during Rotation Stimulation

**Figure 3:**
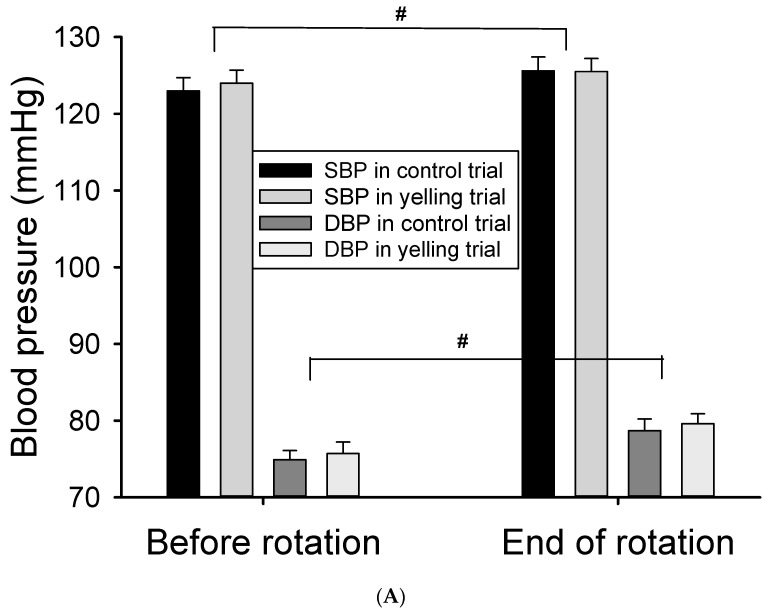

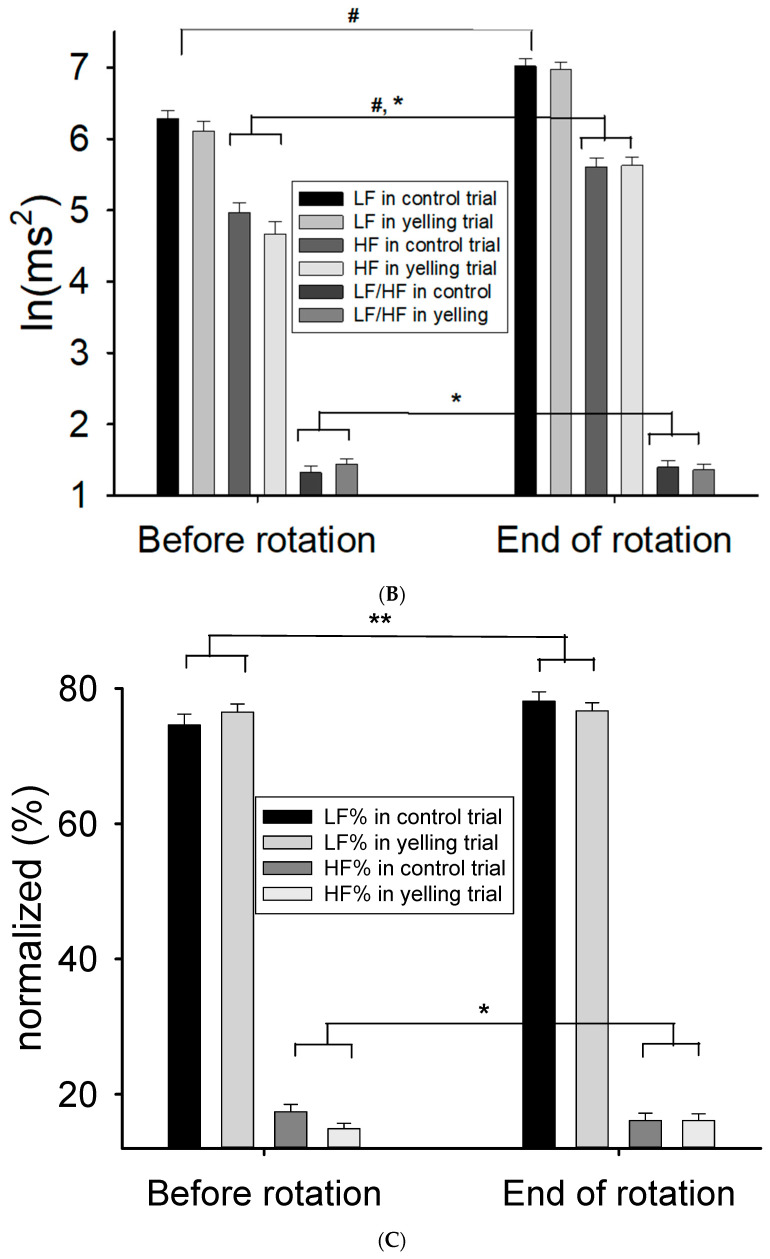
Effect of the yelling intervention on blood pressure and HRV responses during Coriolis stimulation. (**A**) Both systolic blood pressure (SBP) and diastolic blood pressure (DBP) increased significantly after Coriolis stimulation. (**B**) The activities of LF and HF both increase significantly after rotational stimulation, and yelling had a trend of time-by-trial interaction in HF and LF/HF ratio. (**C**) Yelling had a significant time-by-trial interaction in LF%, whereas it had a trend of time-by-trial interaction in HF%. Data are presented as mean ± SEM. # *p* < 0.05, a significant time effect; * 0.05 < *p* < 0.09, a trend of time-by-trial interaction; ** *p* < 0.05, a significant time-by-trial interaction effect.

After rotation stimulation, the heart rate immediately reduced (*p* < 0.001), the blood pressure increased (*p* < 0.05) (Figure 3A), and some HRV activities (including TP, LF, and HF) (main effect of time, *p* < 0.001) increased (Table 3) (Figure 3B). The yelling intervention did not significantly affect cardiovascular response (including heart rate, blood pressure, and most HRV parameters) caused by the 10-min rotation stimulation (trial-by-time interaction). However, it remarkably inhibited LF% after rotation stimulation (*p* = 0.036) (Table 3) (Figure 3C). Moreover, it might reduce sympathetic nervous system activity during motion sickness. In addition, the effect of the yelling intervention on LF/HF ratio (*p* = 0.069), HF (*p* = 0.069), and HF% (*p* = 0.077) in rotational stimulation significantly differed (trial-by-time interaction) (Figure 3B,C). The trend response of the abovementioned HRV parameters might support the notion that yelling could increase parasympathetic nervous system activity (HF), and inhibit sympathetic nervous system activity (HF%, LF/HF ratio) during motion sickness.

We further divided all participants into the susceptible and non-susceptible groups. The effects of the yelling intervention on HRV activity were compared. Results showed that rotation stimulation increased HRV activity (including TP, LF, and HF) in the susceptible and non-susceptible groups (main effect of time, *p* < 0.001) (Table 4). However, the yelling intervention inhibited sympathetic nervous system activity during motion sickness (interaction effect on LF%, *p* = 0.017) only in the non-susceptible group. The activity of all HRV parameters in the susceptible group were not significantly affected by the yelling intervention. In addition, the statistical trend of the yelling intervention affecting LF/HF ratio (*p* = 0.061), HF (*p* = 0.093), and HF% (*p* = 0.074) was observed in the non-susceptible group, but not in the susceptible group.

## 4. Discussion

In this study, in participants who received intermittent rotational stimulation (each lasting for 1 min; 5 times within 10 min) and developed motion sickness, their MSSR score gradually increased with the number of stimulations. However, the yelling intervention immediately improved motion sickness (Figure 2A). This result was extremely significant in the susceptible group, but not in the non-susceptible group, which may be because the symptoms of the non-susceptible group were not obvious. Thus, standardized yelling did not significantly change the symptoms of the non-susceptible group (Figure 2B). Standardized yelling may have practical application value in improving the symptoms of motion sickness susceptible groups. Therefore, its physiological mechanism should be investigated.

This study also performed the d2 Test of Attention before and after rotation stimulation to assess an individual’s cognitive and attention performance (including execution speed, error rate, and correct number) when motion sickness occurred. Moreover, yelling did not significantly affect the scores of cognitive attention during motion sickness. To further confirm the effect of yelling on motion sickness, the degree of attention deficit after rotation stimulation was assessed. Results showed that the score of the d2 Test of Attention was significantly higher after rotation stimulation than before. We hypothesized that this test could effectively detect the effect of yelling on motion sickness, which may be attributed to the influence of two factors. First, the time interval between the two d2 Tests of Attention was extremely short, which led to the learning effect of repeated tests. Thus, the score was significantly higher after rotation stimulation than before. Second, the intensity of the rotational stimulus used was not strong enough to cause a negative effect on cognitive attention. The abovementioned phenomenon was consistent in both the susceptible and non-susceptible groups (Table 1). A reasonable inference should be that the d2 Test of Attention score of the susceptible group should be more negative after the rotation than before, if the rotation stimulus was strong enough. In the future, when considering the intensity of rotation stimulation, in addition to focusing on motion sickness symptoms, the impact of stimulation intensity on cognitive attention should also be considered.

The response to autonomic imbalance caused by motion sickness differs due to the varying methods of inducing motion sickness. Several controversies exist, and there are no definite conclusions. For example, Doweck et al., (1997) stimulated participants with a rotating chair (15 rpm) for 10 min. Results showed that the total power and parasympathetic activity of HRV in individuals susceptible to motion sickness decreased significantly. Nevertheless, it had no evident effects on non-susceptible individuals [25]. In the study of Mullen et al., (1998), moderate motion sickness was induced with the use of a rotating chair (20 rpm) combined with visual stimulation (prism goggles). However, results showed that the HRV did not change significantly, indicating that the autonomic nervous system was not affected by motion sickness [5]. Moreover, Ohyama et al., (2007) used virtual reality stimuli to induce motion sickness, and found that the sympathetic nervous system activity significantly increased. Nevertheless, it did not change the parasympathetic nervous system activity [26]. Yokota et al., (2005) used three-dimensional virtual space visual stimulation to induce motion sickness. Results showed that the sympathetic and autonomic nervous system activity balance ratio (LF/HF) increased significantly, whereas the parasympathetic nervous system activity decreased significantly [6]. In this study, a rotating chair combined with head pitching was used to induce motion sickness. We found that the total power of HRV increased significantly, including a greater parasympathetic nervous system activity (HF power), whereas the sympathetic nervous system activity was only likely to increase (LF%). Thus, there was no significant effect on sympathovagal balance (LF/HF ratio). This result was contrasting to that of a previous study using similar methods for inducing motion sickness [27]. The inconsistency between the two studies may be attributed to the fact that one research required participants to keep their eyes open during rotation, whereas the other study instructed the participants otherwise. Therefore, the addition of visual information may interfere with the autonomic response of simple vestibular stimulation. However, further studies should be conducted to validate the mechanism of its interaction. The current study aimed to assess the practical application of this intervention among pilots. Hence, an experimental design with open eyes should be considered.

This study investigated the effects of standardized yelling on cardiovascular and autonomic responses to validate its physiological mechanism for improving motion sickness symptoms. First, simply yelling at rest was found to increase blood pressure. Nevertheless, it did not affect HRV activities (Table 2). Second, rotational stimulation was associated with a slower heart rate, higher blood pressure, and greater HRV activities (including total power and parasympathetic nervous system activity) (Table 3). In addition, the yelling intervention had a significant impact on HRV only in sympathetic nervous system activity (LF%), which mainly inhibited sympathetic excitement during motion sickness (trial-by-time interaction). However, it did not change blood pressure and heart rate response during motion sickness. The HRV of the susceptible and non-susceptible groups who performed standardized yelling was compared. Results showed that the intervention had a significant inhibitory effect on sympathetic nervous system activity during motion sickness in the non-susceptible group. We hypothesized that the susceptible group has a strong symptom response during motion sickness. Thus, yelling intervention cannot have a significant reversal effect on strong HRV response. The non-susceptible group had less symptoms. Hence, the yelling intervention had a more evident effect on suppressing sympathetic nervous system activity. The inhibitory effect of yelling on sympathetic nervous system activity during motion sickness was consistent with the findings of Chu et al., (2013). The study showed that transcutaneous electrical nerve stimulation (TENS) can significantly reduce sympathetic nervous system activity during motion sickness [28]. Notably, the intervention time of standardized yelling was significantly shorter than TENS (20 min). Thus, it is more convenient in practical applications.

This study had several limitations. First, the participants were not professional pilots, and only ordinary people in good health. Follow-up studies should be performed to assess whether the physiological responses of pilots performing standardized yelling is similar to that of normal healthy people. Second, the yelling maneuvers were only preliminarily standardized in this study, and the practice time and frequency were still limited. Hence, there might still be operational variations. In the future, operational details, such as changes in chest and abdominal pressure caused by the required force exerted by the respiratory muscles for yelling, as well as changes in cardiac output or EEG results, should have more precise specifications to detect the uniformity of learning effects. Third, the methods of measurement of HRV include time domain analysis, frequency domain analysis, and nonlinear metrics. Each of the three methods has its own advantages and disadvantages in research applications [29]. Considering the limitation of research resources and article length, this study only selected frequency domain analysis, which is more sensitive, accurate, and quantitative for short-term analysis. This method seems relatively suitable for the design of this research. However, this study did not compare, analyze, and discuss these three HRV measurement metrics. Fourth, the physiological parameters assessed in this study were limited. Although there was an evident improvement in subjective symptoms, correspondingly significant and intense physiological responses in cardiovascular and autonomic nervous system activities were not observed. Nevertheless, standardized yelling could improve motion sickness symptoms via mechanisms other than the autonomic nervous system. In the future, studies about the effects of motion sickness on EEG should be performed. At present, there are portable real-time monitoring EEG and mental state analysis systems [30,31] that can be used to effectively validate the physiological mechanism of improving motion sickness.

## 5. Conclusions

Standardized yelling can immediately and effectively improve the symptoms of motion sickness in susceptible participants. However, it has a no evident impact on cognitive attention in motion sickness. A yelling maneuver could suppress sympathetic excitability caused by motion sickness, which may be one of the reasons for reducing the symptoms of motion sickness. Hence, the effect of yelling in other physiological mechanisms related to motion sickness (such as EEG) should be further explored. Moreover, in the future, it can be used as a non-pharmacologic measure, as it immediately improves motion sickness symptoms with almost no side effects, thereby maintaining flight safety among pilots and aircrews.

## Figures and Tables

**Table 1:** Effect of the yelling intervention on attention between the susceptible and non-susceptible groups.

	Trials (C vs. Y)	Before Rotation	After Rotation	*p* Value (Trial)	*p* Value (Time)	*p* Value (Trial × Time)
Susceptible group (*n* = 21)						
TN	Control	243.0 ± 15.7	259.4 ± 10.9	0.834	<0.001	0.227
	Yelling	240.6 ± 16.4	265.5 ± 14.4			
E%	Control	33.10 ± 6.04	22.18 ± 4.93	0.714	<0.001	0.621
	Yelling	36.09 ± 7.07	22.78 ± 5.62			
TN-E	Control	176.9 ± 24.8	209.6 ± 17.7	0.848	<0.001	0.205
	Yelling	171.4 ± 26.5	221.9 ± 22.9			
CP	Control	242.6 ± 15.8	259.0 ± 10.9	0.848	0.001	0.205
	Yelling	239.9 ± 16.6	265.1 ± 14.5			
Non-susceptible group (*n* = 21)						
TN	Control	257.8 ± 11.4	284.0 ± 8.8	0.633	<0.001	0.481
	Yelling	250.5 ± 14.9	282.0 ± 11.7			
E%	Control	23.36 ± 4.80	9.78 ± 2.38	0.538	<0.001	0.405
	Yelling	28.40 ± 5.25	10.79 ± 2.39			
TN-E	Control	205.2 ± 17.8	257.8 ± 11.3	0.661	<0.001	0.462
	Yelling	191.0 ± 23.1	254.8 ± 15.7			
CP	Control	256.8 ± 11.5	283.1 ± 8.5	0.662	<0.001	0.460
	Yelling	249.7 ± 14.9	281.6 ± 11.7			

The parameters of the d2 test were TN, E%, TN-E, and CP. TN, total number of items processed; E%, percentage of errors; TN-E, total number of items minus the error scores; CP, concentration performance.

**Table 2:** Effects of simple yelling at rest on various physiologic measurements (*n* = 13).

Parameters	Before Yelling	During Yelling	*p* Value
HR (bpm)	71.8 ± 3.2	70.3 ± 3.3	0.450
SBP (mmHg)	117.1 ± 4.3	121.0 ± 4.4	0.047
DBP (mmHg)	74.5 ± 2.9	79.5 ± 3.3	0.006
TP [ln(ms^2^)]	7.88 ± 0.16	8.02 ± 0.17	0.169
LF [ln(ms^2^)]	7.27 ± 0.15	7.35 ± 0.15	0.377
HF [ln(ms^2^)]	5.95 ± 0.19	6.04 ± 0.24	0.747
LF/HF (ln ratio)	1.32 ± 0.08	1.36 ± 0.16	0.747
LF (%)	77.13 ± 1.60	76.74 ± 2.49	0.852
HF (%)	17.15 ± 1.05	16.98 ± 2.02	0.934

HR = heart rate; SBP = systolic blood pressure; DBP = diastolic blood pressure. The parameters of heart rate variability were TP, LF, HF, LF/HF, LF%, and HF%.

**Table 3:** Effects of the yelling intervention on various physiologic responses in rotation stimulation (*n* = 42).

Parameters	Trials	Before Rotation	End of Rotation	*p* Value (Trial)	*p* Value (Time)	*p* Value (Trial × Time)
HR (bpm)	Control	74.8 ± 1.6	71.7 ± 1.4	0.858	<0.001	0.236
	Yelling	75.6 ± 1.7	71.3 ± 1.7			
SBP (mmHg)	Control	123.0 ± 1.7	125.6 ± 1.8	0.724	0.019	0.423
	Yelling	124.0 ± 1.7	125.5 ± 1.7			
DBP (mmHg)	Control	74.9 ± 1.2	78.7 ± 1.5	0.351	<0.001	0.947
	Yelling	75.7 ± 1.5	79.6 ± 1.3			
TP [ln(ms^2^)]	Control	6.94 ± 0.11	7.67 ± 0.09	0.848	<0.001	0.202
	Yelling	6.84 ± 0.12	7.73 ± 0.09			
LF [ln(ms^2^)]	Control	6.29 ± 0.11	7.02 ± 0.10	0.315	<0.001	0.372
	Yelling	6.11 ± 0.14	6.98 ± 0.09			
HF [ln(ms^2^)]	Control	4.97 ± 0.14	5.61 ± 0.12	0.228	<0.001	0.069
	Yelling	4.67 ± 0.17	5.63 ± 0.11			
LF/HF (ln ratio)	Control	1.32 ± 0.09	1.40 ± 0.09	0.607	0.979	0.069
	Yelling	1.44 ± 0.07	1.36 ± 0.08			
LF (%)	Control	74.6 ± 1.6	78.1 ± 1.4	0.826	0.098	0.036
	Yelling	76.5 ± 1.2	76.7 ± 1.2			
HF (%)	Control	17.4 ± 1.1	16.1 ± 1.1	0.131	0.947	0.077
	Yelling	14.9 ± 0.8	16.1 ± 1.0			

HR = heart rate; SBP = systolic blood pressure; DBP = diastolic blood pressure. The parameters of heart rate variability were TP, LF, HF, LF/HF, LF%, and HF%**.**

**Table 4:** Effects of the yelling intervention on HRV activity in rotation stimulation between the susceptible and non-susceptible groups.

Parameters	Trials	Before Rotation	End of Rotation	*p* Value (Trial)	*p* Value (Time)	*p* Value (Trial × Time)
Susceptive group (*n* = 21)						
TP [ln(ms^2^)]	Control	6.97 ± 0.17	7.67 ± 0.14	0.588	<0.001	0.691
	Yelling	7.02 ± 0.15	7.76 ± 0.15			
LF [ln(ms^2^)]	Control	6.32 ± 0.17	6.96 ± 0.17	0.906	<0.001	0.917
	Yelling	6.33 ± 0.15	6.99 ± 0.15			
HF [ln(ms^2^)]	Control	4.99 ± 0.22	5.55 ± 0.18	0.942	<0.001	0.508
	Yelling	4.94 ± 0.18	5.58 ± 0.18			
LF/HF (ln ratio)	Control	1.33 ± 0.10	1.42 ± 0.12	0.788	0.583	0.538
	Yelling	1.40 ± 0.09	1.41 ± 0.12			
LF (%)	Control	74.9 ± 1.8	78.1 ± 1.7	0.998	0.164	0.567
	Yelling	75.6 ± 1.8	77.4 ± 1.8			
HF (%)	Control	16.9 ± 1.5	15.3 ± 1.4	0.249	0.333	0.590
	Yelling	15.2 ± 1.1	14.6 ± 1.4			
Non-susceptible group (*n* = 21)						
TP [ln(ms^2^)]	Control	6.90 ± 0.14	7.67 ± 0.11	0.412	<0.001	0.229
	Yelling	6.65 ± 0.19	7.71 ± 0.11			
LF [ln(ms^2^)]	Control	6.25 ± 0.14	7.07 ± 0.11	0.122	<0.001	0.360
	Yelling	5.89 ± 0.23	6.98 ± 0.12			
HF [ln(ms^2^)]	Control	4.94 ± 0.16	5.68 ± 0.15	0.103	<0.001	0.093
	Yelling	4.41 ± 0.27	5.67 ± 0.14			
LF/HF (ln ratio)	Control	1.31 ± 0.14	1.39 ± 0.13	0.650	0.535	0.061
	Yelling	1.48 ± 0.09	1.30 ± 0.11			
LF (%)	Control	74.2 ± 2.6	78.2 ± 2.4	0.772	0.381	0.017
	Yelling	77.5 ± 1.5	76.0 ± 1.6			
HF (%)	Control	17.9 ± 1.7	16.8 ± 1.7	0.327	0.389	0.074
	Yelling	14.6 ± 1.2	17.6 ± 1.4			

HR = heart rate; SBP = systolic blood pressure; DBP = diastolic blood pressure. The parameters of heart rate variability were TP, LF, HF, LF/HF, LF%, and HF%.

## Data Availability

The data in this study are collected and owned by the Aviation Physiology Research Laboratory, Taiwan, and cannot be shared publicly due to the regulations.
